# PPO Inhibitors as a Key Focus in Herbicide Discovery

**DOI:** 10.3390/molecules31081270

**Published:** 2026-04-12

**Authors:** Min Zhao, Baojian Li, Ying Gao, Rui Zhang, Subinur Ahmattohti, Jie Li, Xinbo Shi

**Affiliations:** 1 School of Pharmacy, Xinjiang Second Medical College, Karamay 834000, China; 18232189381@163.com (M.Z.); lbj9591@163.com (B.L.); 18829190996@163.com (Y.G.); zhangrui@xjsmc.edu.cn (R.Z.); 18016910278@163.com (S.A.); 2 Science and Innovation Center of Xinjiang Second Medical College, Karamay 834000, China; 3 Shaanxi Collaborative Innovation Center of Chinese Medicine Resources Industrialization, Shaanxi University of Chinese Medicine, Xianyang 712046, China

**Keywords:** PPO inhibitors, herbicide discovery, molecular design

## Abstract

As the key enzyme catalyzing the final step in heme and chlorophyll biosynthesis, protoporphyrinogen oxidase (PPO) is a crucial target for herbicide development. To date, over 40 PPO inhibitors have been commercialized. They offer high efficacy, environmental safety, low application rates, and broad-spectrum weed control. Recently, significant progress has been made in PPO structural biology, with several crystal structures resolved. Despite decades of use, the emergence of resistant weeds necessitates the continuous innovation of novel PPO inhibitors. This review systematically summarizes PPO three-dimensional structures, enzyme-inhibitor interaction mechanisms, and quantitative structure–activity relationships (QSARs). Finally, we outline rational molecular design strategies for the next generation of PPO inhibitors.

## 1. Introduction

**Figure 1:**
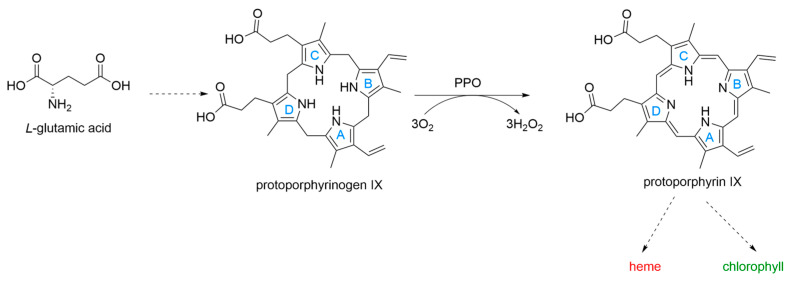
Protoporphyrinogen oxidase (PPO) catalyzes the enzymatic reaction converting protoporphyrinogen IX (the scaffolds can mainly be divided into skeletons A, B, C, and D) to protoporphyrin IX, the penultimate step in porphyrin biosynthesis. Solid arrows indicate single enzymatic steps, dashed arrows indicate multiple enzymatic steps.

Protoporphyrinogen oxidase (PPO; EC 1.3.3.4) serves as the final shared catalytic component within the biosynthetic routes that produce both chlorophyll and heme [1]. This membrane-associated enzyme family exhibits high evolutionary conservation and is present across diverse taxa, including bacteria, fungi, plants, and mammals. During the reaction, PPO facilitates the six-electron oxidation of protoporphyrinogen-IX into the fully conjugated macrocycle known as protoporphyrin IX in a molecular oxygen-dependent environment (Figure 1). Within plant systems, PPO exists as two distinct isoforms: the mitochondrial PPO2 and the plastid PPO1 [2]. Specifically, PPO1 is integrated into the chloroplast envelope and thylakoid membranes, whereas PPO2 is localized on the outer surface of the inner mitochondrial membrane.

The metabolic disorder known as variegated porphyria (VP) is a dominant hereditary condition arising from pathogenic variations within the human PPO gene. Clinically, VP is characterized by neurological manifestations, acute abdominal distress, and dermal photosensitivity [3,4,5]. This condition exhibits a higher prevalence among females than males and can manifest abruptly at any life stage, ranging from adolescence to late adulthood [6]. Research indicates that patients diagnosed with VP typically demonstrate a minimum 50% reduction in PPO enzymatic activity [7]. Such enzymatic impairment, often triggered by specific amino acid substitutions near the active site (notably the R59W mutation), results in the systemic accumulation of protoporphyrin IX, thereby heightening light sensitivity in affected individuals.

Furthermore, scientific focus on PPO has expanded considerably following the discovery of its therapeutic potential in cancer management via photodynamic therapy (PDT) [8]. Specifically, Halling and colleagues [9] established that PPO inhibitors can induce the targeted buildup of photosensitizing protoporphyrinogen-IX within malignant cells.

In botanical science, PPO is recognized as a critical molecular target for a wide range of structurally heterogeneous herbicides [10]. This diverse group encompasses chemical families such as diphenylethers [11], phenylpyrazole [12], oxadiazoles [13], triazolinones [14], thiadiazoles [15], pyrimidindiones [16], oxazolidinediones [17], and *N*-phenylphthalimides [18], among various other classes [19].

The pharmacological interference with this plant enzyme initiates a pathological cascade, beginning with the intracellular surge of the substrate protoporphyrinogen-IX. This accumulated precursor is subsequently translocated to the cytosol, where it undergoes spontaneous, non-catalytic oxidation driven by molecular O_2_ within the vicinity of mitochondria and chloroplasts [20]. This reaction yields a photo-reactive variant of protoporphyrin IX. Upon exposure to solar radiation, this intermediate facilitates the generation of singlet oxygen, a highly reactive species that precipitates lethal lipid peroxidation and eventual cellular collapse. Consequently, these PPO-targeting compounds are formally categorized as light-dependent bleaching herbicides [21].

The present review aims to synthesize the prevailing understanding of PPO structural characteristics across various species, specifically focusing on human, bacterial, and botanical sources. Furthermore, it elucidates the complex binding modalities between PPOs and their inhibitors, alongside an exploration of the quantitative structure–activity relationships (QSARs) that define these chemical interactions. Finally, the article details the rational molecular design principles that have underpinned the development of contemporary commercial PPO inhibitors.

## 2. Structure of PPOs

**Figure 2:**
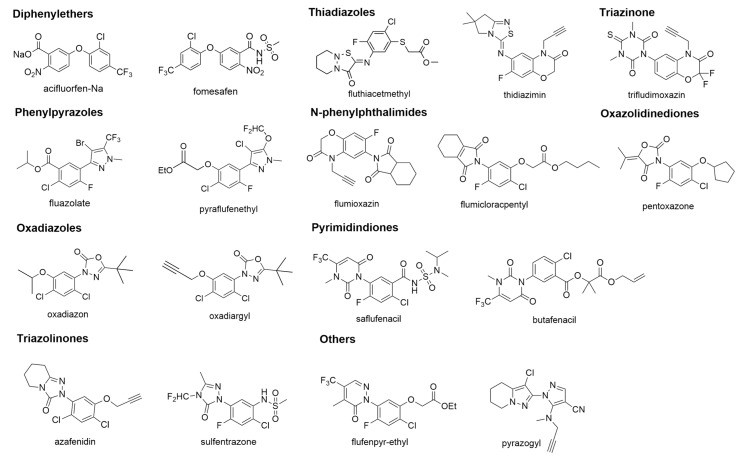
Chemical structures of selected representative protoporphyrinogen oxidase-inhibiting herbicides.

Deciphering the spatial architecture of an enzyme is essential for elucidating catalytic pathways, mapping inhibitor-enzyme associations, and facilitating rational drug discovery. In 2004, the inaugural crystal structure of tobacco-derived mitochondrial PPO2 (mtPPO) was characterized by Koch et al. [22], revealing its complex with the inhibitor INH (fluazolate, see Figure 2). This dimeric, yellow-pigmented protein was resolved at 2.9 Å using selenium-based single anomalous diffraction. Its molecular scaffold comprises three distinct regions: an α-helical domain responsible for membrane attachment; a FAD-binding domain resembling the topology of *p*-hydroxybenzoate hydroxylase; and a substrate-binding domain housing a constricted active site beneath the FAD cofactor.

Subsequent crystallographic studies expanded this structural library to include bacterial PPO from Myxococcus xanthus (mxPPO) [23], Bacillus subtilis (bsPPO) [24], and the human ortholog (hPPO) [25], typically studied in complex with acifluorfen (Figure 2). While mtPPO, mxPPO, and hPPO function as membrane-associated dimers sensitive to diphenylether herbicides, bsPPO represents a distinct outlier within this enzyme family. Notably, bsPPO is a soluble, monomeric protein that possesses a more expansive substrate range and exhibits resistance to AF inhibition.

Sequence alignment reveals that mtPPO, mxPPO, bsPPO, and hPPO exhibit minimal primary sequence identity. Nevertheless, these enzymes share remarkably congruent global folding patterns, as characterized by Koch et al. [22]. Their structural framework consistently integrates three functional domains: an FAD-binding domain, a substrate-binding domain, and a membrane-binding domain. Specifically, the FAD-binding region demonstrates substantial structural and sequence homology with the broader flavoenzyme family.

The high degree of structural conservation is further evidenced by the superimposition of the mtPPO substrate-binding domain onto those of mxPPO, bsPPO, and hPPO, yielding root-mean-square deviations (RMSD) for C_α_ atoms of 0.7 Å, 1.1 Å, and 0.8 Å, respectively. Apart from minor variations in specific loop regions—likely stemming from evolutionary insertions or deletions—the conformations of the substrate-binding and FAD-binding domains across these four enzymes are nearly indistinguishable [23,24,25].

In stark contrast, the membrane-binding domains display significant conformational divergence, which is presumably dictated by their distinct lipid-interaction mechanisms. For instance, crystallographic analysis suggests that mtPPO associates with the membrane as a homodimer [22], whereas mxPPO does not form a physiologically active dimeric state. Instead, for mxPPO, the hydrophobic interface comprising helices 4, 5, and 10 within its membrane-binding domain is proposed to function as a specialized membrane anchor [23].

**Figure 3:**
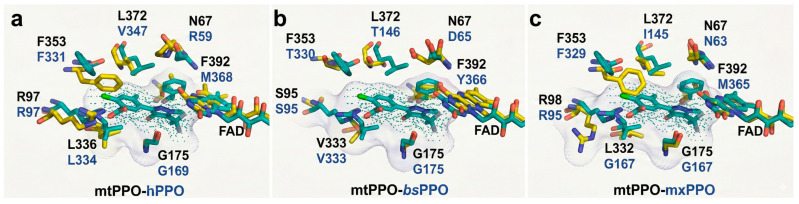
The active sites of PPOs. (**a**) Mitochondrial and human PPOs (mtPPO-hPPO); (**b**) mitochondrial and bacterial PPOs (mtPPO-bsPPO); (**c**) Mitochondrial and bacterial PPOs (mtPPO-mxPPO).

The PPO catalytic center is a hydrophobic pocket situated at the junction of the substrate-binding and FAD-binding domains, housing a suite of functionally significant and evolutionarily conserved residues [22,23,24,25]. As illustrated in Figure 3, the most invariant of these is a glycine residue (identified as Gly175 in mtPPO, Gly167 in mxPPO, Gly169 in hPPO, and Gly175 in bsPPO). The carbonyl oxygen of this glycine extends from the base of the pocket toward the center of the active site, where it facilitates interactions with the tetrapyrrole macrocyclic framework.

Another prominent conserved residue is the arginine located at positions 98, 95, and 97 in mtPPO, mxPPO, and hPPO, respectively; notably, this position is occupied by Ser95 in the bsPPO ortholog. Based on substrate-bound structural simulations, this residue is thought to engage in ionic interactions or hydrogen bonding with the C-ring propionic acid group. The consistent presence of this bulky side chain on one side of the binding pocket appears to impose steric constraints on the opposing side, which invariably accommodates smaller, uncharged residues such as Ala, Gly, Thr, or Ser. Furthermore, a third highly conserved site is occupied by Phe353 (corresponding to Phe329 in mxPPO, Phe331 in hPPO, and Thr330 in bsPPO). This residue is strategically positioned to define the vertical clearance of the cavity ceiling relative to the FAD macrocycle.

Figure 3 further delineates additional pivotal residues within the active site which, despite lacking high evolutionary conservation, exhibit comparable steric or chemical characteristics across diverse PPO species. A notable example is the stacking interaction with ring A of protoporphyrinogen-IX, mediated by Phe392 in mtPPO [22], a position occupied by Met365 and Tyr366 in mxPPO and bsPPO, respectively. Similarly, the position adjacent to the FAD isoalloxazine ring shows significant variability: the Asn67 found in mtPPO is replaced by Asn63 in mxPPO, Asp65 in bsPPO, and Arg59 in hPPO.

This human-specific Arg59 has garnered extensive research interest due to its indispensable role in sustaining enzymatic functionality and structural equilibrium in vitro. Before the definitive resolution of the hPPO crystal structure, computational models based on mtPPO suggested that Arg59 formed a critical salt bridge with Asp349, thereby preserving the active site’s structural framework. Among various pathological variants, the R59W mutation is the most frequently analyzed, given its strong correlation with Variegated Porphyria (VP). Crystallographic evidence presented by Qin et al. [25] reveals that the hydrophilic binding environment actively repels the hydrophobic indolyl ring of the substituted tryptophan. This repulsion severely undermines the necessary interaction between the FAD isoalloxazine ring and the incoming substrate.

Additionally, the binding pocket utilizes a pair of variable residues to stabilize the substrate’s B ring through hydrophobic “sandwiching.” This structural motif is formed by Leu356 and Leu372 in mtPPO, Leu332 and Ile345 in mxPPO, Val333 and Thr346 in bsPPO, and Leu334 and Val347 in hPPO.

Notably, the diversity in substrate selectivity observed across various PPO orthologs is primarily attributed to significant variations in the dimensions of their respective binding pockets. Investigations by Qin and colleagues [24,25] quantified the internal volumes of these cavities, reporting 1173 Å^3^ for bsPPO, 440 Å^3^ for mtPPO, and 627 Å^3^ for mxPPO. Consequently, the pocket within bsPPO is capable of housing substrates that are twofold to threefold larger than those accommodated by either mtPPO or mxPPO. Furthermore, distinct electrostatic properties were identified within the bsPPO substrate-binding chamber, where the interior surface is characterized by a lining of positive charges—a feature noticeably absent in the binding environments of mtPPO and mxPPO. These combined structural and electronic factors likely underpin the enhanced substrate versatility characteristic of bsPPO relative to other members of the PPO enzyme family.

Currently, the definitive binding configuration of protoporphyrinogen-IX remains elusive, primarily because a crystal structure of PPO complexed with its native substrate or related structural analogs has yet to be resolved. Utilizing the mtPPO-inhibitor (INH) complex as a structural proxy, Koch and associates [22] formulated the initial comprehensive binding model for the substrate. Their findings suggest that the two anionic propionyl moieties are oriented toward the solvent-accessible regions of the pocket, with the C-ring propionyl group establishing an ionic bridge with the invariant Arg98. Furthermore, the A ring participates in π-π stacking with Phe392, while the B ring is sequestered between the conserved residues Leu356 and Leu372.

This spatial arrangement aligns the C20 methylene bridge—which connects the A and D rings—in close proximity to the reactive N5 position of the FAD cofactor, supporting the hypothesis of a triphasic oxidation pathway [22]. Furthermore, understanding the kinetic parameters of PPOs is vital for elucidating their catalytic efficiency and the precise nature of their inhibition. The enzymatic conversion of protoporphyrinogen IX to protoporphyrin IX involves a complex six-electron oxidation process. Kinetic studies across different species have reported varying Michaelis-Menten constants K_m_ and turnover numbers k_cat_, with the catalytic efficiency k_cat_/K_m_ reflecting the enzyme’s affinity and processing speed for the native substrate. Additionally, the sequence of substrate binding and product release—specifically whether the multi-step electron transfer follows a ping-pong bi-bi mechanism or involves a ternary complex with molecular oxygen—remains a critical subject of investigation. Determining these kinetic frameworks is essential, as it clarifies whether a given inhibitor acts competitively against the initial substrate association, interferes with an intermediate oxidation step, or blocks final product dissociation. To better illustrate these specific enzymatic properties, the kinetic parameters of wild-type and selected mutant PPOs are summarized in Table 1 [26,27].

A central feature of this proposed mechanism is that hydride abstraction consistently occurs at the C20 position of the tetrapyrrole framework, mediated by hydrogen rearrangements via imine-enamine tautomerization. Although this model has gained broad consensus, several fundamental ambiguities persist. For instance, while protoporphyrinogen-IX undergoes spontaneous oxidation by O_2_, the rate-limiting step of this non-catalytic transformation—and the precise divergence between enzymatic and non-enzymatic pathways—remains to be elucidated. Additionally, it remains undetermined whether the overall reaction rate is governed by substrate association or product dissociation kinetics.

Existing inhibitors typically function by emulating specific structural motifs of the native substrate, thereby engaging in competitive binding. Intriguingly, these agents employ diverse modes of molecular mimicry, and even a single inhibitor may exhibit ortholog-specific binding configurations across different species. For instance, INH serves as a structural analog for rings A and B of protoporphyrinogen-IX, with its carboxylate moiety effectively substituting for the propionate group attached to ring C [22].

Crystallographic evidence further highlights this variability; acifluorfen replicates the conformation of rings A and B in both mxPPO and hPPO [23,24], yet it aligns with the geometry of rings C and D when associated with bsPPO [25]. These observations imply that the combined binding profiles of two acifluorfen molecules can encompass the entire tetrapyrrole framework of the substrate.

Crucially, these structural and mechanistic insights directly guide the rational design of novel herbicides. For example, the invariant Arg98 serves as a universally prime anchoring point; incorporating strong hydrogen-bond acceptors (such as specific carbonyl or sulfonyl groups) into the inhibitor scaffold can effectively lock the molecule into this site. Similarly, the unique electrostatic landscape and significantly larger pocket volume of bacterial orthologs like bsPPO inspire the design of bulkier, “dual-mimicry” molecules. By exploiting these distinct steric and electronic environments, researchers can develop highly targeted, species-selective inhibitors or novel architectures that overcome target-site resistance mutations.

## 3. PPO Inhibitors as a Key Focus in Herbicides

### 3.1. Herbicide Classes and Inhibition Characteristics

Research into PPO herbicides has persisted for over six decades, though their specific pharmacological mechanisms remained obscure until the mid-1980s. These existing inhibitors typically function by emulating specific structural motifs of the native substrate, protoporphyrinogen IX, thereby engaging in competitive binding at the active site and specifically blocking the multi-step oxidation process. Compared to alternative weed-control agents, PPO inhibitors present numerous strategic advantages, such as minimal toxicological impact and exceptional efficacy at modest application rates (10–50 g.ai/ha). Furthermore, they demonstrate a comprehensive herbicidal spectrum encompassing both monocotyledonous and dicotyledonous species, alongside a rapid onset of activity—often inducing necrosis within 24 h—and sustained residual effectiveness.

Owing to these environmentally benign characteristics, several structural classes have been successfully commercialized over the past several decades. These include diphenylethers [11], phenylpyrazole [12], oxadiazoles [13], triazolinones [14], thiadiazoles [15], pyrimidindiones [16], oxazolidinediones [17], and *N*-phenylphthalimides [18]. Representative examples of these commercial PPO herbicides are detailed in Figure 2 [11]. All of them are competitive inhibitors in the mechanism.

### 3.2. Resistance Mechanisms

In comparison to various other herbicidal categories, the emergence of resistance to PPO herbicide has proceeded at a notably protracted pace. Despite their introduction to the commercial market in the 1960s, documented resistance has been confined to few weed species to date [28]. Among these, *Amaranthus tuberculatus*—a primary agricultural threat in the Midwestern United States—was first identified in 2001 as exhibiting cross-resistance to both PPO and acetohydroxamic synthase (AHAS) inhibitors. This resistance profile was subsequently observed in three additional dicot species: *Euphorbia heterophylla*, *A. quitensis*, and *Ambrosia artemisiifolia*.

Interestingly, the rise in PPO resistance is partially linked to the prior management of AHAS-resistant populations [29], as practitioners pivoted to PPO inhibitors to control weeds that no longer responded to AHAS-targeting chemistries. For instance, certain *A. tuberculatus* biotypes now resist PPO inhibitors such as lactofen and fomesafen, alongside AHAS inhibitors like imazethapyr and chlorimuron-ethyl. Patzoldt et al. [30] identified that resistance within natural *A. tuberculatus* populations is conferred by a specific Gly210 codon deletion in the PPX2L gene, which targets both mitochondrial and plastid PPO isoforms. This mechanism is particularly distinctive because it involves an amino acid deletion rather than the more typical substitution found in target-site resistance.

**Figure 4:**
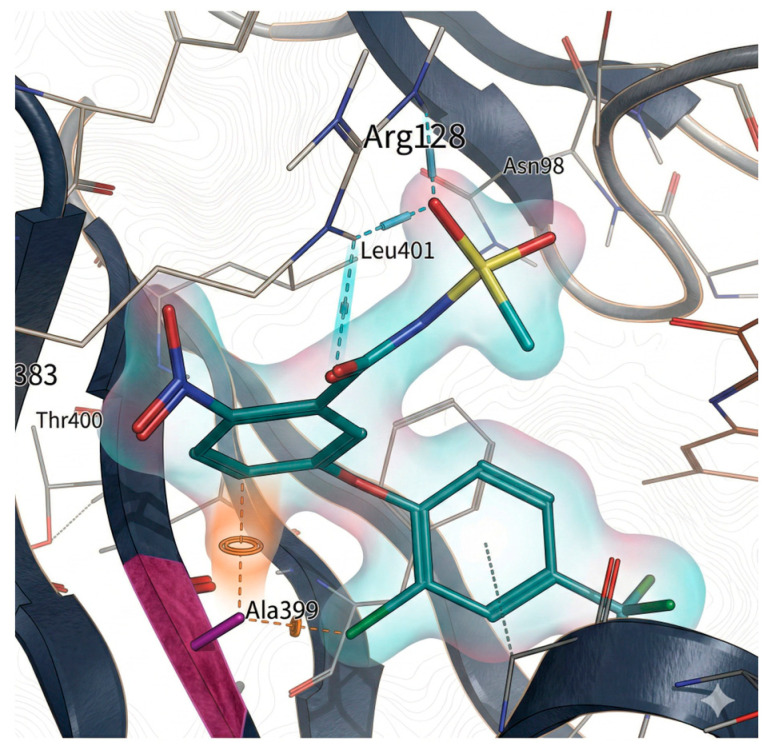
Position of the G399A mutation relative to the predicted binding mode of fomesafen. Fomesafen (cyan) was modeled into the binding site of a homology model of a PPO2 protein sequence with G399A (gray) mutation. The alanine mutation is shown in pink [31].

Recent research by Burgos et al. [31] identified a novel G399A mutation in the PPO2 catalytic domain of *Amaranthus palmeri* that confers broad resistance to PPO-inhibiting herbicides. Computational modeling and biochemical assays revealed that this glycine-to-alanine substitution creates steric hindrance, reducing herbicide binding affinity (see Figure 4). Although less prevalent in field populations than the ΔG210 mutation [32], G399A endows significant cross-resistance to multiple herbicide classes, including diphenylethers and triazolinones.

### 3.3. Development of Resistant Crops

The engineering of crops resistant to PPO inhibitors has recently emerged as a focal point of intense scientific inquiry [1,33,34]. Research strategies have primarily centered on traditional tissue culture methodologies, the manipulation of co-factor expression for protoporphyrin IX-binding subunit proteins, and the deliberate overproduction of the endogenous plant PPO gene. To date, successful instances of herbicide tolerance have been documented in several cultivars, including rice, maize, tomato, tobacco, and soybean [28], with resistance levels spanning a broad spectrum from 2-fold to 1000-fold.

Owing to the structural heterogeneity inherent in this herbicide class, the advancement of resistance technologies does not rely upon a solitary herbicide or a specific mutant gene. For instance, investigative efforts have explored herbicide-clearance mechanisms, such as chelatase-based approaches and the utilization of P-450 monooxygenases [35]. While the P-450 monooxygenase pathway holds promise for developing herbicide-resistant germplasm, its application to PPO-targeting chemistries faces a significant hurdle: the rapid physiological onset of these herbicides. Consequently, standard metabolic detoxification may be insufficiently fast to counteract such rapid-acting compounds. To address this, future engineering must prioritize P-450 monooxygenases characterized by exceptional catalytic efficiency and the versatile capacity to metabolize a diverse array of PPO inhibitor structures.

## 4. Quantitative Structure–Activity Relationships of PPO Inhibitors

Following the patenting of oxadiazon and chlorophthalim in the early 1970s, a vast array of molecules has been synthesized and scrutinized for their herbicidal efficacy and PPO inhibitory potential. To elucidate the underlying interaction mechanisms and streamline structural refinement, various Quantitative Structure–Activity Relationship (QSAR) models have been established [36]. A comprehensive synthesis of these QSAR advancements was provided by Fujita et al. [37], covering diverse chemical families such as thiadiazoles, diphenylethers, *N*-phenylphthalimides, and various *N*-phenyl-substituted heterocycles.

**Figure 5:**
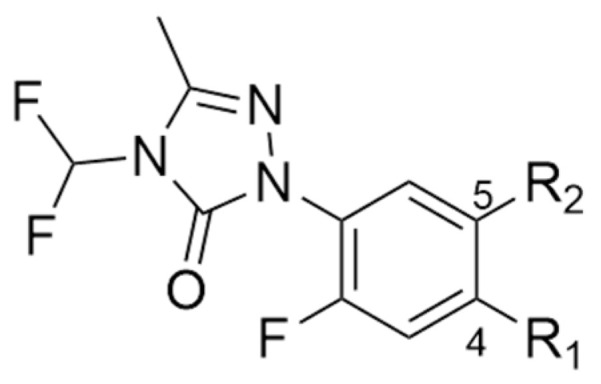
Structure and herbicidal activity of *N*-phenyl triazolinone derivatives.

Notably, the predictive accuracy and robustness of a QSAR equation are fundamentally contingent upon the strategic selection of molecular descriptors as well as the size and representativeness of the dataset. A prominent case in point is the *N*-phenyltriazolinone class (see Figure 5), a significant group of PPO inhibitors pioneered by researchers at the FMC Corporation. This category includes key commercial agents such as sulfentrazone, a pre-emergent herbicide for soybean crops, and carfentrazone-ethyl, utilized for post-emergent weed control in cereals. Theodoridis and colleagues [14] conducted a targeted QSAR evaluation on a library of 1-substitutedphenyl-4H-1,2,4-triazolin-5-ones, utilizing hydroponic cucumber seedling assays to measure biological potency. The study investigated the correlation between herbicidal activity pI_50_ and two primary physicochemical parameters: the hydrophobicity constant π and the STERIMOL steric term B_1_. Specifically, fourteen derivatives featuring substituents at the R_2_ position (position 5) of the phenyl ring were analyzed using Equation (1):

(1) $$pI_{50}=7.02\left(\pm 2.351\right)B_{1}-0.18\left(\pm 0.045\right)\pi^{2}-2.57\left(\pm 0.775\right)B_{1}^{2}+2.51$$

This model successfully explained 78% of the biological variance (r^2^ = 0.78). However, it is critical to acknowledge that this model relies on a highly restricted dataset of only 14 compounds, which significantly limits its predictive robustness, reproducibility, and applicability across broader chemical spaces. From a chemical perspective, the model suggests that activity reaches a peak when B_1_ = 1.35 and π = 0.02. This indicates that the optimal inhibitor must strike a delicate physicochemical balance: it requires specific spatial dimensions (dictated by B_1_) to accommodate the steric constraints of the active site, while maintaining an ideal lipophilicity (π) that facilitates leaf membrane permeation and root uptake. Despite these insights, divergent findings were later presented by Nicolaus et al. [38], whose subsequent QSAR modeling suggested that herbicidal efficacy was predominantly governed by substituent hydrophobicity rather than steric factors. This discrepancy further underscores the limitations of classical QSAR models when reliant on small datasets and basic semiempirical parameters.

To overcome these descriptive limitations, Density Functional Theory (DFT) has evolved into an indispensable instrument for precisely estimating geometric configurations and electronic profiles. A pioneering illustration of this application is the research conducted by Xi et al. [39], who introduced the inaugural quantum-chemical descriptor specifically designed to quantify steric effects. By modeling the volumetric distribution of the electron cloud via DFT simulations, they established a QSAR model with exceptional predictive fidelity for 35 sulfonylurea derivatives. It is significant to highlight that, while a multitude of quantum-chemical parameters had already been successfully integrated into QSAR frameworks, a dedicated theoretical metric for representing spatial hindrance had remained elusive until this development.

Zhang and colleagues [40] broadened the utility of the DFT-QSAR framework by applying it to the prediction of bioactive molecular arrangements. Their investigation concentrated on a library of cyclic imide analogs, for which three independent QSAR models were constructed, each corresponding to a distinct equilibrium conformation characterized via DFT descriptors.

Building upon these foundational SAR principles and other pioneering works [41,42], recent research [43] has applied Multivariate Image Analysis (MIA) to model the inhibitory activity of a diverse set of 61 benzothiazole derivatives. This advanced methodology encodes specific atomic properties—namely, Pauling’s electronegativity (ε), van der Waals radii (r_vdw_), and their ratio (r_vdw_/ε)—directly into color-coded 2D chemical images. By utilizing a genetic algorithm to select the most relevant descriptors, researchers constructed highly robust Multiple Linear Regression (MLR) models for rapid activity prediction. For instance, the MLR model based on the r_vdw_/ε ratio is defined by the equation:

(2) $$pK_{i}=12.7253\left(\pm 1.3219\right)+0.0029\left(\pm 0.0014\right)\times MIA\left(r_{vdW}/\epsilon \right)5574\;+0.0013\left(\pm 0.0003\right)\times MIA\left(r_{vdW}/\epsilon \right)9348\;+0.0011\left(\pm 0.0002\right)\times MIA\left(r_{vdW}/\epsilon \right)5561\;-0.0171\left(\pm 0.0036\right)\times MIA\left(r_{vdW}/\epsilon \right)6022\;+0.0006\left(\pm 0.0003\right)\times MIA\left(r_{vdW}/\epsilon \right)2692$$

These MLR models, alongside complementary Partial Least-Squares (PLS) models, demonstrated exceptional statistical reliability, achieving correlation coefficients (r^2^) between 0.85 and 0.88, cross-validation scores (q^2^) of 0.75 to 0.83, and external predictive abilities (r^2^_pred_) ranging from 0.77 to 0.86 (Table 2).

**Figure 6:**
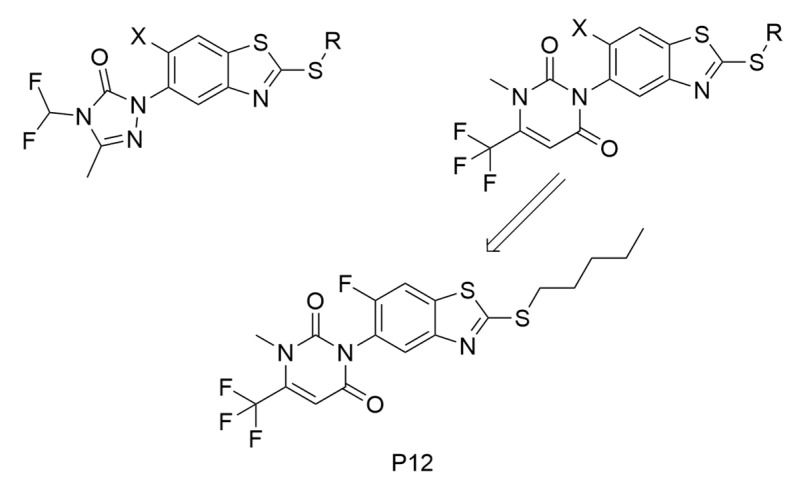
benzothiazole derivatives and the lead compound **P12**.

The structural insights derived from these MIA-QSAR contour maps and variable importance in projection (VIP) scores clearly identified critical regions for molecular optimization. Specifically, highly electronegative halogens like fluorine at the benzothiazole’s X position significantly enhance binding affinity compared to chlorine or bromine. Additionally, incorporating longer alkyl chains at the R position improves hydrophobic interactions within the PPO active site. Leveraging these computational insights, a novel lead compound named **P12** (Figure 6) was conceptualized. **P12** features a pyrimidine heterocycle coupled with a pentyl chain, yielding a predicted activity (pK_i_ = 7.93) that rivals the most potent known derivatives. Crucially, **P12** boasts a calculated lipophilicity (log P = 5.45) substantially higher than that of commercial standards like sulfentrazone (log P = 4.55). This elevated lipophilicity not only implies improved leaf membrane permeation for post-emergence efficacy but also suggests a lower potential for environmental leaching, firmly establishing MIA-QSAR as a powerful paradigm for discovering the next generation of safe and effective agricultural herbicides.

Notably, the configuration indicated by the DFT-QSAR approach demonstrated exceptional structural alignment with the validated bioactive form. Consequently, this methodology serves as a streamlined alternative for determining the bioactive orientations of small molecules, proving particularly advantageous in scenarios where the tertiary structure of the target protein remains uncharacterized. This synergy between quantum mechanical calculations and quantitative structure–activity modeling significantly pushes the traditional boundaries of classical QSAR disciplines.

The preceding evidence underscores those descriptors derived from DFT yield predictive models with higher accuracy than those based on semiempirical methods or traditional physicochemical parameters. A particularly salient advantage of the DFT-QSAR framework is its ability to resolve the bioactive conformations of small molecules.

Despite the historical success and structural insights provided by classical and DFT-based QSAR models, the contemporary landscape of agrochemical discovery is rapidly shifting toward more sophisticated computational paradigms. Traditional QSAR approaches are often bottlenecked by limited datasets and linear assumptions that struggle to capture the highly complex, non-linear realities of protein-ligand interactions. In recent years, the integration of machine learning (ML) and artificial intelligence (AI) has dramatically expanded the horizons of herbicide discovery [44,45,46]. Modern AI-driven algorithms, when coupled with large-scale virtual screening and structure-based design strategies (such as high-throughput molecular docking and molecular dynamics simulations), offer far superior predictive power and validation rigor compared to isolated QSAR equations. These modern pipelines allow researchers to virtually evaluate millions of structurally diverse scaffolds, predict target-site mutation resistance, and optimize multi-parameter ADMET (Absorption, Distribution, Metabolism, Excretion, and Toxicity) profiles simultaneously. Consequently, while traditional QSAR descriptors remain valuable for intuitive chemical interpretation, future breakthroughs in designing “resistance-breaking” PPO inhibitors will inevitably rely on the seamless integration of experimental structural biology with advanced machine-learning architectures.

## 5. The Recent Evolution of PPO Inhibitors

The distinct attributes associated with PPO herbicides have garnered significant interest from the global agrochemical community. Over the past ten years, substantial research endeavors have been directed toward the development of novel PPO inhibitors, leading to the identification of several noteworthy candidates that exhibit exceptional herbicidal potency.

The protoporphyrinogen-IX molecule exhibits a bipartite architecture dictated by its pyrrole ring substituents. This structure is characterized by a distinct lipophilic domain on one side, while the opposing side, bearing the propionic acid side chains, presents a significantly more hydrophilic profile. Although it was traditionally assumed that herbicides compete with the substrate by mimicking only two of its pyrrole rings, a major advancement occurred with the design of an inhibitor specifically engineered to replicate the spatial arrangement of the three rings [47], but has not been commercially launched to date [12].

Beyond the triazolinone framework, various other heterocyclic scaffolds were extensively explored. Following the recognition of phenyluracils’ herbicidal potential in the late 1980s, researchers successfully substituted the triazolinone moiety with a dihydropyrimidine-2,4-dione ring, establishing an entirely new class of phytotoxic agents [48]. A critical refinement in this series was the introduction of a trifluoromethyl group at the 6-position of the ring, which substantially enhanced biological potency. This optimization trajectory eventually led to the commercialization of four pivotal herbicides: flupropacil, benzfendizone, butafenacil, and saflufenacil [49] (see Figure 2).

Among these uracil-based compounds, saflufenacil has been registered as a highly effective selective herbicide for broadleaf weed management. It is versatile enough for both pre-emergence and post-emergence regimes across several key crops—including cotton, sunflowers, and soybeans—as well as for use in non-agricultural and fallow environments. Notably, saflufenacil demonstrates exceptional efficacy, providing a robust solution for controlling weed populations that have evolved resistance to glyphosate and AHAS-inhibiting chemistries.

Given their extensive weed-control profile, novel uracil-centered PPO inhibitors serve as compelling templates for scaffold hopping and isosteric investigations aimed at discovering new lead motifs to manage resistant biotypes, which have moved beyond classical templates toward “resistance-breaking” architectures and pivoted toward increased structural complexity. Such as from traditional Two-Ring to Three-Ring architectures, Bioisosteric Side-Chain Replacement.

**Figure 7:**
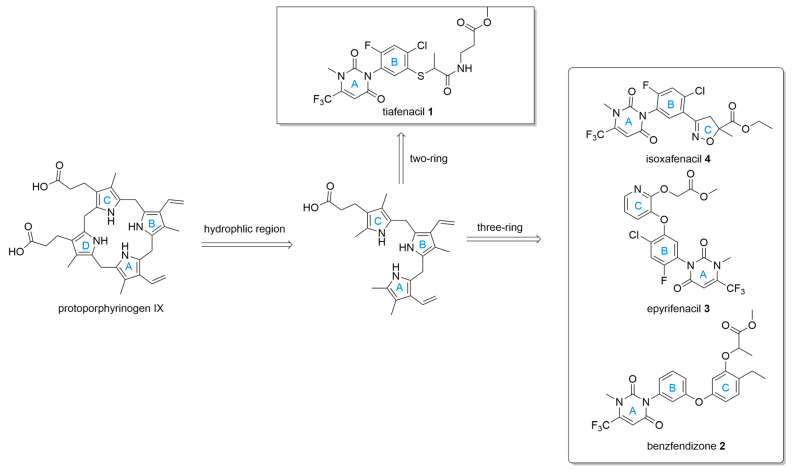
Novel uracil-centered PPO herbicides with two-ring or three-ring. Their scaffolds/mimics can be mainly divided into the corresponding skeletons A, B, C, and D.

The first successfully commercialized uracil-centered herbicide with a two-ring system after saflufenacil is tiafenacil (Figure 7, compound **1**). Studies [50] have elucidated the biochemical and physiological mode of action of tiafenacil. In vitro assays utilizing recombinant PPO enzymes from diverse plant species, including amaranth, soybean, arabidopsis, and rapeseed, demonstrated that tiafenacil possesses a highly potent binding affinity, with half-maximal inhibitory concentration IC_50_ values ranging narrowly from 22 to 28 nM. This high level of enzymatic inhibition is comparable to other highly active pyrimidinediones like saflufenacil and butafenacil, as well as the *N*-phenylphthalimide herbicide flumioxazin. Furthermore, tiafenacil proved to be significantly more efficient than traditional diphenyl ether (DPE) herbicides, exhibiting 3- to 134-fold lower IC_50_ values compared to fomesafen, oxyfluorfen, and acifluorfen.

The physiological consequences of this PPO inhibition follow a robust, light-dependent mechanism. When plant tissues are treated with tiafenacil in the dark, the enzymatic blockade leads to a marked accumulation of protoporphyrin IX. Upon subsequent exposure to light, this accumulation triggers a severe chain reaction of cellular damage. This oxidative stress is quantitatively confirmed by a rapid decrease in the Fv/Fm values of chlorophyll fluorescence, indicating compromised photosystem II efficiency, alongside a simultaneous increase in malondialdehyde (MDA) content, which serves as a biological marker for extensive lipid peroxidation and membrane disruption. As a result of this rapid structural degradation, tiafenacil acts as a highly effective, broad-spectrum herbicide capable of controlling both dicotyledonous and monocotyledonous plants at applied concentrations between 1 and 50 µM. While dicot weeds like velvetleaf and amaranth are extremely sensitive and face lethal desiccation at doses as low as 1 to 5 µM, monocots such as barnyardgrass and rice show slightly higher natural tolerance but are still effectively controlled at higher concentrations.

The recent publication by Sada et al. [51] introduces epyrifenacil (Figure 7, compound **3**), a novel herbicide developed by Sumitomo Chemical that represents a significant evolution in the pyrimidinedione class of PPOs. Epyrifenacil is characterized by a unique three-ring structure that incorporates a pyridine ring. Unlike traditional contact-based PPO inhibitors, epyrifenacil exhibits exceptional systemic activity. Upon absorption, it is rapidly converted into an active acid metabolite that translocates efficiently through the plant’s phloem in both basipetal and acropetal directions. This systemic movement allows for the effective control of a broad spectrum of both broadleaf and grass weeds at exceptionally low application rates, such as 20 g a.i. ha^−1^. Furthermore, the herbicide demonstrates significant efficacy against weed biotypes that have evolved target-site resistance to existing PPO inhibitors, successfully controlling problematic species like Palmer amaranth carrying the ΔG_210_, Arg128Gly, or Gly399Ala mutations. In addition to its potent broad-spectrum and systemic herbicidal activity, epyrifenacil possesses an extremely low vapor pressure, which minimizes the risk of off-target drift and ensures the safety of adjacent sensitive crops.

To address the limitations of traditional PPOs, which typically exhibit weaker control over grassy weeds, researchers [52] designed a novel class of uracil-isoxazoline derivatives by integrating the highly active uracil scaffold of saflufenacil with an isoxazoline moiety. Utilizing the intermediate derivatization method, a series of nineteen new compounds were synthesized and evaluated for their herbicidal efficacy. Structure–activity relationship (SAR) analysis revealed that an ethyl substituent paired with a methyl group (identified as compound **4**, Figure 7) provided the optimal configuration for broad-spectrum activity. This optimized derivative demonstrated exceptional post-emergence activity at application rates as low as 7.5 g/ha, effectively controlling both broadleaf weeds and notoriously difficult grassy weeds, significantly outperforming saflufenacil on the latter. Furthermore, the compound exhibited potent efficacy against glyphosate-resistant biotypes, including Conyza canadensis and Eleusine indica, and produced a remarkable synergistic effect when applied in combination with glyphosate, highlighting its significant potential as a next-generation PPO-inhibiting herbicide for broad-spectrum weed management. Zhen Xi et al. [41,42,53,54,55] also developed a series of compounds via the “intermediate derivatization method,” including *N*-Phenylaminomethylthioacetylpyrimidine-2,4-diones, N-Phenylisoxazoline-thiadiazolo[3,4-a]pyridazine, and others, but no commercialization has been reported to date.

Alnafta et al. [56] developed a series of novel uracil-based PPOs. The research leveraged scaffold hopping and bioisosteric replacement strategies, focusing specifically on the structural diversification of the side chains found in established inhibitors like tiafenacil and epyrifenacil. By utilizing molecular modeling based on a wild-type Amaranthus tuberculatus crystal structure, the authors designed and synthesized unprecedented side-chain motifs, including thioisoxazolines, thiolactams, and thioacrylamides. Experimental results indicate that isoxazoline-based derivatives provide effective control over resistant Amaranthus populations. Furthermore, the introduction of a thioacrylamide side chain represents a significant breakthrough, affording exceptional efficacy against resistant monocotyledonous weeds like ryegrass (*Lolium* spp.) and black-grass (*Alopecurus myosuroides*). These advancements not only broaden the herbicidal spectrum but also offer improved crop safety in corn and wheat, illustrating that bioisosteric refinement is a pivotal tool for sustaining the utility of the PPO-inhibitor class.

Besides the commercialized and upcoming molecules mentioned above, researchers [57] have also developed other molecules through active substructure linking and bioisosterism replacement strategies to design a novel series of 47 tetrahydrophthalimide derivatives incorporating oxadiazole and thiadiazole moieties. By substituting these heterocycles at the 5-position of the *N*-phthalimide benzene ring, the study identified a compound featuring a 1,3,4-oxadiazole ring as a highly potent lead structure. In vitro assays demonstrated that this compound possesses an exceptional binding affinity for Nicotiana tabacum PPO (NtPPO) with a K_i_ of 9.05 nM, significantly outperforming the commercial standard flumiclorac-pentyl (K_i_ = 46.02 nM). Molecular docking and molecular dynamics simulations revealed that this enhanced affinity is driven by strong hydrogen bonding with the Arg98 (2.9 Å) and Ser235 (2.7 Å) residues, alongside stable π-π stacking with Phe392 and π-conjugation with Leu356 and Leu372.

## 6. Conclusions

In summary, more than six decades of research have firmly established PPO as a highly effective and validated target for herbicide discovery. PPO-inhibiting herbicides possess favorable traits, such as high efficacy at low application rates and environmental friendliness, though improper application can lead to crop injury. Although a wide array of PPO inhibitors have been commercialized, the escalating evolution of weed resistance—particularly driven by target-site mutations like ΔG210 and G399A—continues to fuel the urgent need for developing next-generation PPO-inhibiting herbicides.

Moving forward, the discovery of robust PPO inhibitors will heavily depend on the seamless integration of structural biology with emerging computational technologies. While refining chemical structures via innovative pharmacophores (such as scaffold hopping, bioisosteric replacement, and pro-herbicide activation) remains essential, the incorporation of artificial intelligence (AI) and machine learning (ML) models into virtual screening pipelines will be transformative. These advanced tools offer an unprecedented ability to rapidly traverse vast chemical spaces, predict ADMET profiles, and specifically design molecules capable of accommodating resistant mutated active sites.

Furthermore, fundamental questions concerning PPO structure and function must still be addressed to fully unlock the target’s potential. These include elucidating the detailed kinetic mechanisms of the enzyme, mapping the precise sequence of the six-electron oxidation, and understanding the exact dynamics of product release. Finally, because all current commercial PPO inhibitors function through competitive inhibition, exploring allosteric or non-competitive inhibition represents a highly promising and critically underexplored alternative strategy to systematically overcome existing resistance mechanisms.

## Figures and Tables

**Table 1:** Selected Kinetic parameters of wild-type and mutant PPOs.

PPO Type	Protein Mutants	Km (μM)	Kcat (min^−1^)	Kcat/Km (min^−1^/μM)
human PPO	Wild-type	2.08 ± 0.11	2.97 ± 0.15	1.43
	R168S (Substrate ring A)	11.33 ± 0.93	3.32 ± 0.22	0.29
	V170T (Substrate ring B)	2.97 ± 0.31	7.70 ± 0.66	2.59
	L166N (Substrate ring C)	5.13 ± 0.71	1.82 ± 0.13	0.35
	R97G (Substrate ring D)	19.64 ± 2.14	76.37 ± 9.15	3.89
*N. tabacum* PPO2	Wild-type	1.17	6	5.1
	F392E (Substrate ring A)	11.20	11	1.0
	L372N (Substrate ring B)	16.40	7	0.4
	R98E (Substrate ring C)	12.50	34	2.7
	S374D (Substrate ring D)	10.9	208	18.5

**Table 2:** The robust and predictive coefficients for MLR models.

Model Descriptor Type	Fitting (r^2^)	Cross-Validation (q^2^)	External Prediction (r^2^_pred_)
r_vdw_	0.865	0.808	0.818
ε	0.861	0.792	0.866
r_vdw_/ε	0.872	0.827	0.775

## Data Availability

Data sharing is not applicable to this article as no new data were created. The crystal structure data discussed in this review are available in the Protein Data Bank (PDB) under the accession codes cited in the text and figure legends.

## Abbreviations

The following abbreviations are used in this manuscript:

AI	artificial intelligence
bsPPO	Bacillus subtilis PPO
DFT	Density Functional Theory
FAD	flavin adenine dinucleotide
hPPO	human ortholog PPO
MD	molecular dynamics
MDA	malondialdehyde
MIA	Multivariate Image Analysis
ML	machine learning
MLR	Multiple Linear Regression
mtPPO	mitochondrial PPO2
mxPPO	Myxococcus xanthus PPO
NtPPO	Nicotiana tabacum PPO
NMR	nuclear magnetic resonance
PDT	photodynamic therapy
PLS	Partial Least-Squares
PPO	protoporphyrinogen oxidase
QSARs	quantitative structure–activity relationships
SAR	structure–activity relationships
VIP	Variable Importance in Projection
VP	variegated porphyria
